# Reducing contrast-agent volume and radiation dose in CT with 90­kVp tube voltage, high tube current modulation, and advanced iteration algorithm

**DOI:** 10.1371/journal.pone.0287214

**Published:** 2023-06-15

**Authors:** Min Su Park, Hong Il Ha, Jhii-Hyun Ahn, In Jae Lee, Hyun Kyung Lim

**Affiliations:** 1 Department of Radiology, Hallym University Sacred Heart Hospital, Anyang-si, Gyeonggi-do, Republic of Korea; 2 Department of Radiology, Yonsei University Wonju College of Medicine, Wonju Severance Christian Hospital, Wonju, Gangwon-do, Republic of Korea; 3 Department of Radiology, Soonchunhyang University Seoul Hospital, Seoul, Republic of Korea; Chung-Ang University Gwangmyeong Hospital, REPUBLIC OF KOREA

## Abstract

Increasing utilization of computed tomography (CT) has raised concerns regarding CT radiation dose and technology has been developed to achieve an appropriate balance between image quality, radiation dose, and the amount of contrast material. This study was planned to evaluate the image quality and radiation dose in pancreatic dynamic computed tomography (PDCT) with 90­kVp tube voltage and reduction of the standard amount of contrast agent, compared with 100­kVp PDCT of the research hospital’s convention. Total of 51 patients with both CT protocols were included. The average Hounsfield units (HU) values of the abdominal organs and image noise were measured for objective image quality analysis. Two radiologists evaluated five categories of image qualities such as subjective image noise, visibility of small structure, beam hardening or streak artifact, lesion conspicuity and overall diagnostic performance for subjective image quality analysis. The total amount of contrast agent, radiation dose, and image noise decreased in the low-kVp group, by 24.4%, 31.7%, and 20.6%, respectively (*p* < 0.001). The intraobserver and interobserver agreements were moderate to substantial (*k* = 0.4−0.8). The contrast-to-noise ratio (CNR), signal-to-noise ratio (SNR), and figure of merit of the almost organs except psoas muscle in the low-kVp group were significantly higher (*p* < 0.001). Except for lesion conspicuity, both reviewers judged that subjective image quality of the 90-kVp group was better (*p* < 0.001). With 90­kVp tube voltage, 25% reduced contrast agent volume with advanced iteration algorithm and high tube current modulation achieved radiation dose reduction of 31.7%, as well as better image quality and diagnostic confidence.

## Introduction

The rapidly increasing utilization of computed tomography (CT) has raised concerns regarding potential hazards due to CT radiation [1]. In conjunction with radiation hazards, CT technology has been developed to achieve an appropriate balance between image quality, reduction in radiation dose, and the amount of contrast material [2,3]. The following technological advances, such as a decrease in tube potential and an increase in compensatory tube current as well as iterative reconstruction (IR), have contributed the most [4,5]. Low-tube-voltage imaging causes increased image noise and susceptibility to beam hardening artifacts, simultaneously resulting in degradation of image quality [6]. The aforementioned disadvantage of low-tube-voltage imaging is compensated by IR. IR not only improves the overall image quality by reducing image noise but also contributes to improved diagnostic confidence [6–8]. Based on these advantages, low tube voltage and IR techniques have been widely used in CT scans in children, CT angiography, and chest CT [9–13].

However, low-tube-voltage imaging is limited in adult abdominal CT. A decrease in radiation penetration resulting from low tube voltage may lead to an increase in image noise and create a blotch image artifact in solid organs, such as the liver, resulting in image quality degradation [14,15]. Low-tube-voltage imaging shows higher iodine contrast enhancement because the mean photon energy approaches the iodine k­edge of 33 keV [16,17]. Higher iodine contrast enhancement is a double-edged sword in adult abdominal CT. It improves the contrast-to-noise ratio (CNR) and signal-to-noise ratio (SNR) of the lesion and each abdominal organ. However, enhanced local radiation effect and increased beam hardening artifacts around the contrast agent-filled vessels, such as the aorta, splenic artery, or superior mesenteric artery occurs, which may affect the evaluation of the adjacent pancreas [18,19]. As mentioned earlier, when the tube voltage is reduced, the X-ray photons become less penetrating, resulting in reduced attenuation by the patient’s body, which can also lead to increased image noise due to the lower SNR. To compensate for this limitation, two briefly aforementioned techniques are commonly used. The first is high-tube current modulation, an advanced automatic exposure control technique that adjusts the tube current during the scan to optimize image quality and reduce radiation dose. By increasing the number of X-ray photons that reach the detector, high tube current modulation can improve image quality via improving image contrast. The second technique is IR, which is an advanced image processing technique that can reduce image noise while preserving image detail [20,21]. Thus, feasible CT imaging in the adult abdominal region using low tube voltage and IR has been gradually reported.

The reduction in the amount of iodine contrast material is another major concern in CT image acquisition because contrast material-induced nephrotoxicity is closely related to pre-existing renal insufficiency and the total amount of contrast materials [22]. We hypothesized that a higher iodine contrast enhancement increased by low tube voltage imaging can be adjusted to a level that does not affect the image quality and diagnosis by reducing the total amount of contrast medium. Therefore, the purpose of the study was to evaluate the image quality and radiation dose reduction between the 90-kVp pancreatic dynamic CT (PDCT) with less than the standard amount of contrast agent and the 100-kVp PDCT with the standard amount of contrast agent.

## Materials and methods

This retrospective study was conducted in accordance with the Helsinki declaration of ethical principles for medical research involving human subjects and approved by the Institutional Review Board and Ethics Committee of Hallym University Sacred Heart hospital with the requirement for written informed consent was waived and confirmed. Design of the study followed the Strengthening the reporting of observational studies in epidemiology (STROBE) guidelines (S1 Table).

### Sample size estimation and patient enrollment

The study was designed for matched pairwise comparison. The minimum sample size was determined to be 44 patients under the assumption of effect size of 0.5, a statistical significance of 0.05 and a power of 90%.

**Fig 1:**
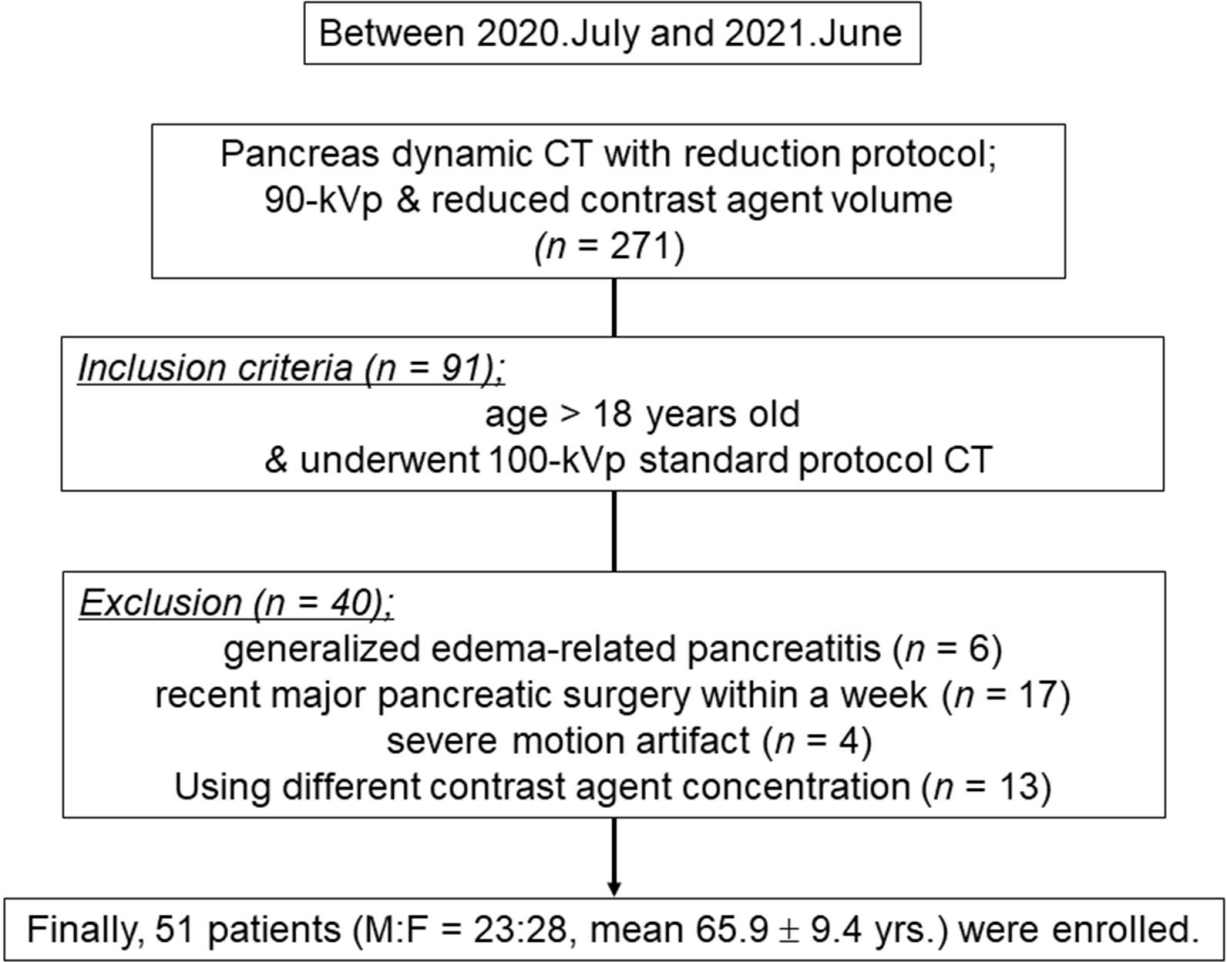
Patient selection flowchart. From July 2020 to June 2021, 271 patients were eligible for the study, and according to the inclusion and exclusion criteria, final 51 subjects were selected as the study population.

From July 2020 to June 2021, two hundred seventy one patients received 90­kVp tube voltage and reduced contrast agent administration protocol. Among them, ninety-one patients above 18 years of age received both 90­kVp and 100­kVp tube voltage PDCT within the same period. Forty patients were excluded by following reasons: severe motion artifacts (n = 1), marked generalized edema-related pancreatitis (n = 6), recent pancreatic surgery within a week (n = 17), or different iodine contrast agent concentration (n = 13). Ultimately, 51 patients were enrolled in the study (Fig 1).

### CT protocol

All CT images were obtained using one of three machines (SOMATOM Force, SOMATOM Definition Edge, SOMATOM Definition Flash, Siemens Healthineers). Among them, SOMATOM Force employed a Tin filter, which absorbs high-energy X–rays and allows only low-energy X-rays to be used for patient exposure, helping to reduce the radiation dose during CT scans. The reduction protocol group was scanned with 90­kVp tube voltage and 374 mAs or 393 mAs of CT vendor’s reference tube current time product using MDCT scanners (SOMATOM Force, Siemens Healthineers) with a reduction of the standard amount of contrast material (300 mg of iodine per mL, approximately 1.4 ml/kg, according to the dosage adjustment table (S2 Table)) and reconstructed by advanced IR algorithm (ADMIRE strength 2, Br40 of the reconstruction kernel). The standard protocol group was scanned with a 100­kVp tube voltage and 289 mAs of CT vendor’s reference tube current time product using two MDCT scanners (SOMATOM Definition Edge or SOMATOM Definition Flash; Siemens Healthineers) using a standard amount of contrast material (300 mg of iodine per mL, 2 ml/kg) and reconstructed using the IR algorithm (SAFIRE strength 2, I40 of reconstruction kernel). PDCT consists of a dual phase (arterial and portal venous phases). All patients received an intravenous nonionic contrast medium containing an iodine concentration of 300 mg/mL (Bonorex^®^ 300 (iohexol), Central Medical Service, Seoul, Korea; Iomerol^®^ 300 (iomeprol), Bracco Imaging, Seoul, Korea). For dynamic imaging, nonionic contrast material per kilogram of body weight was administered at a rate of 2­5 mL/s using an automatic power injector (Multilevel CT, Medrad, Warrendale, Pennsylvania, USA), followed by a 20 mL flush of sterile saline. Bolus tracking method was used to determine the timing of arterial phase scanning, and the arterial phase was obtained 15 seconds after triggering when the proximal abdominal aorta reached the Hounsfield unit (HU) of 100 or greater. Portal venous phase images were acquired 30 seconds after the end of arterial phase scan. The reconstruction parameters of the axial image were a 3 mm section thickness and a 3 mm reconstruction interval. A coronal reformatted image was reconstructed with a section thickness of 3 mm and an interval of 3 mm. Table 1 is comparison of both CT acquisition protocols.

**Table 1:** Comparison of CT scan protocol, radiation dose and contrast agent volume reduction.

	Reduction protocol group (90-kVp tube voltage & reduced contrast agent volume)	Standard protocol group (100-kVp tube voltage & standard dose of contrast agent)	*p*-value
Age	65.9 ± 9.4 (41−90)		-
Sex (male: female)	23: 28		-
Height (cm)	159.5 ± 9.4 (143.0−177.0)		-
Weight (kg)	62.9 ± 11.2 (39.8−99.7)		-
Body mass index	24.6 ± 3.1 (18.7−33.4)		-
CT interval day)	203.8 ± 128.4 (27−336)		-
CT scanner	Somatom Force	Somatom Definition Edge or Flash	-
kVp	Sn90-kVp	100-kVp	-
Reference tube current time product (mAs)	289 mAs	374−393 mAs	< 0.001
Effective tube current time product (mAs)	245.9 ± 69.5	174.2 ± 64.0	< 0.001
Contrast material volume (ml)	95.5 ± 17.0 (60−135)	126.3 ± 22.3 (100−164)	< 0.001
CTDIvol (arterial phase)	7.3 ± 2.7 (4.8−16.5)	10.5 ± 4.1 (4.6−21.4)	< 0.001
CTDIvol (portal phase)	7.0 ± 2.2 (4.7−14.8)	10.1 ± 4.0 (4.2−19.1)	< 0.001
Effective diameter (sum, cm)	54.6 ± 4.9 (44.3−67.7)		−
Size­specific dose estimate (mGy)	9.5 ± 2.3 (5.5−16.0)	13.9 ± 4.8 (4.7−23.1)	< 0.001
Iteration technique & strength	ADMIRE, strength 2	SAFIRE, strength 2	
Reconstruction kernel	I40	Br40	
Slice thickness & interval	3 mm & 3 mm	3 mm & 3 mm	

Numbers in parentheses indicate ranges.

### Radiation dose evaluation

The radiation dose of the pre-contrast images was excluded from the evaluation because the kVp of the pre-contrast scan was not fixed. The only radiation dose in the portal phase images was compared. The size-specific dose estimate (mGy∙cm) was calculated by multiplying the value of volume CT dose index (CTDIvol) with the appropriate conversion factors (f). The conversion factor (f) depends on the diameter of the patient using the sum of the anteroposterior and transverse dimensions according to the report 204 released by the American Association of Physicists in Medicine (AAPM) [23]. The effective tube current time product (mAs) of each patient in the portal phase was recorded.

### Image analysis

#### Objective assessment of image quality

The standard deviation (SD) of the air located outside the patient’s xyphoid process level was defined as the objective image noise, which is also used as the background image noise. The mean CT HU of each abdominal organ, such as the aorta, liver, main portal vein (MPV), pancreas, spleen, kidney, and psoas muscle, as well as cystic lesions of the pancreas and liver which is more than 5 mm in diameter, were measured using a circular region of interest. When drawing region of interests (ROIs) in each target organ, special care was taken not to include adjacent vessels, bile ducts, artefacts, or peritoneal fat.

The mean CT HU of the aorta, pancreatic parenchyma, and psoas muscle were measured in both the arterial and portal phases. The mean CT HU values of the liver, MPV, spleen, and kidney were measured only during the portal phase of CT scanning. Liver attenuation was recorded as the mean of the measurements of four ROIs in the medial and lateral segments of the left hepatic lobe, and the anterior and posterior segments of the right hepatic lobe [7]. Aortal attenuation was measured at the celiac trunk take-off level. The attenuation of the psoas muscle was recorded as the mean attenuation of two ROIs that avoided macroscopic fat infiltration at the L4 vertebral level. Kidney attenuation was measured in the renal cortex, with special care taken to avoid containing the medulla and perirenal fat. The size, shape, and position of all ROI measurements were kept constant by applying a copy-and-paste function at the workstation. The SNR and CNR were calculated as follows:

$$SNR_{target\;organ}=HU_{target\;organ}/background\;image\;noise$$

$$CNR_{target\;organ}=(HU_{target\;organ}-HU_{psoas\;muscle})/background\;image\;noise$$

Figure of Merit of the aorta, liver, MPV, pancreas, kidney, spleen, and image noise was compared to characterize the performance of both protocols using the following equation:

$$Figure\;of\;Merit_{target\;organ}={\left(SNR_{target\;organ}\right)}^{2}/SSDE$$

#### Subjective image quality analysis

The subjective image quality analysis was independently and blindly evaluated by two board-certified radiologists with more than 10 years of experience. One reviewer was from an off-site institution. Subjective image noise, beam hardening or streak artefacts, visibility of small structures (peripheral hepatic vessels), lesion conspicuity, and overall diagnostic confidence were evaluated using a 5­point scale based on the European Guidelines on Quality Criteria for Computed Tomography and previous research published in the radiology literature (S3 Table) [24–26]. All 102 PDCT sets were reviewed by two reviewers without any information of patient and scan technique. Subjective image noise was graded on a 5­point scale based on the presence and amount of image mottle or graininess (5, minimal image noise; 4, less than average noise; 3, average image noise; 2, above average noise; 1, unacceptable image noise). The visibility of small structures, mainly hepatic vessels, was also graded using a 5-point scale, with 5 indicating excellent visualization and 1 indicating imperceptible small hepatic vessel structures. Beam hardening or streak artifacts were graded on a 5­point scale (5, absence of artifact; 4, mild artifacts not interfering with diagnosis; 3, moderate artifacts slightly interfering with diagnosis; 2, pronounced artifacts interfering with diagnosis; and 1, impossible interpretation of a lesion or an organ of interest).

Hepatic cysts, pancreatic cystic lesions, and hepatic hemangiomas more than 5mm in short diameter were selected by an independent researcher for lesion conspicuity evaluation. Each reviewer received brief lesion information including image number on arterial or portal phase, anatomic location, or adjacent anatomic landmark.

Lesion conspicuity was ranked on a 5­point scale with a score of 5 indicating a clearly seen lesion with clearly visualized margins and a score of 1 indicating an imperceptible lesion. Lesion conspicuity was evaluated based on the visibility of the lesion boundary. When more than 75% of the boundary of the lesion was visible, it was marked as 5 points; when 50%­75% of the boundary was visible, it was marked as 4 points; when 25­50% of the boundary was visible, it was marked as 3 points; and when only less than 25% of the boundary was visible, it was marked as 2. Overall diagnostic confidence was evaluated using a 5­point scale; grade 1, non­confident; grade 2, sub­diagnostic confidence; grade 3, average confidence; grade 4, more than average; and grade 5, completely confident.

### Statistics

Continuous variables are expressed as means and SD. Intra­observer and interobserver agreements were assessed using the weighted kappa statistics. Quantitative image parameters (attenuation values, image noise, SNR, and CNR) and the figure of merit were corrected using Welch’s test depending on normality testing and compared using paired *t-tests*. Qualitative subjective image analysis of the image was performed using the Wilcoxon signed-rank test. Statistical analysis was performed using the MedCalc software (Version 13.1.2, MedCalc, Ostend, Belgium). Statistical significance was set at *p* < 0.05.

## Results

### Patient demographics and radiation dose

**Fig 2:**
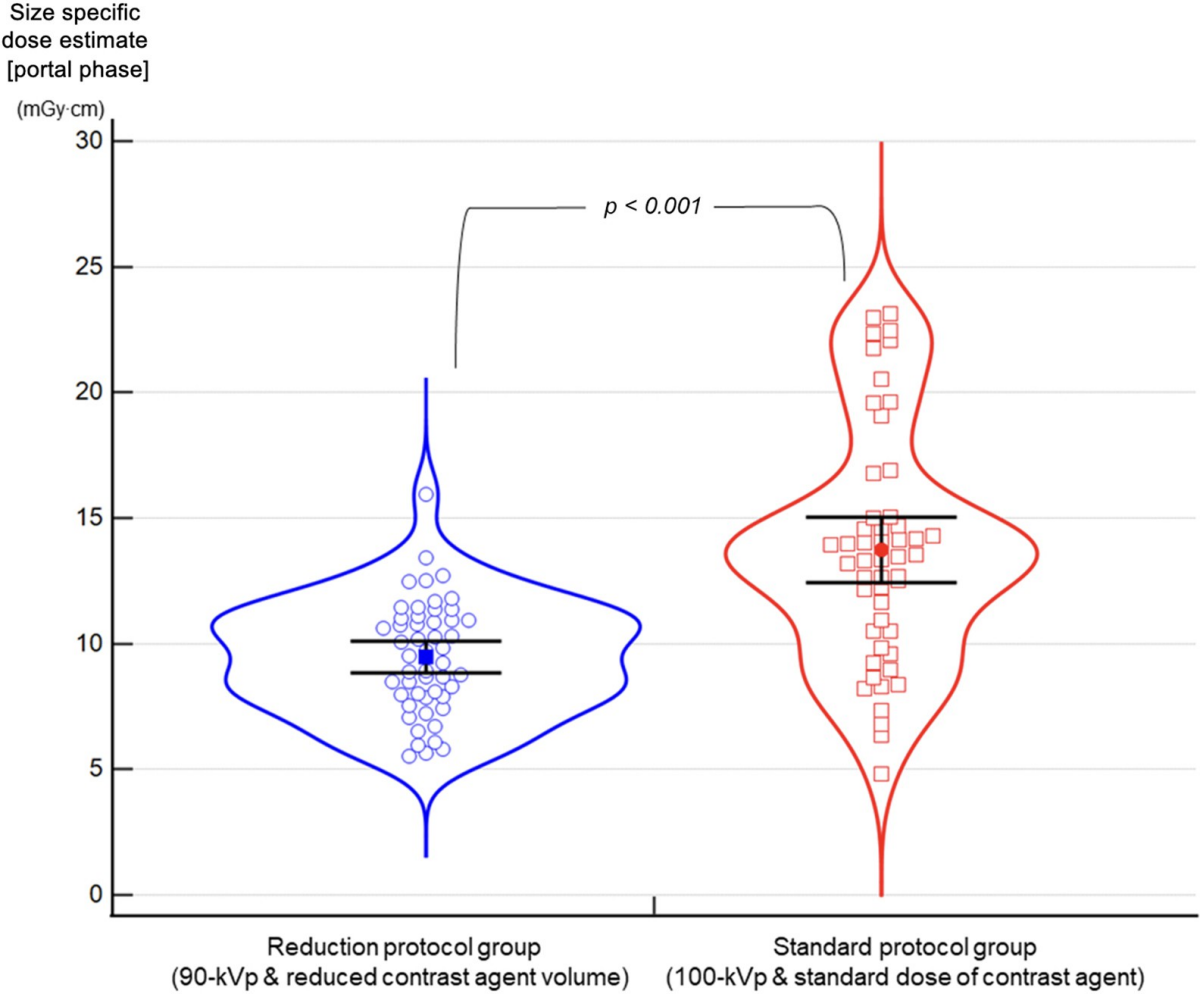
Violin plot comparison of size-specific dose estimation. The blue graph represents the reduction protocol group, while the red graph represents the standard protocol group in the violin plot chart. The Y-axis represents the size-specific dose estimate of the portal phase. The blue empty circles represent the size-specific dose estimate of each individual in the reduction protocol group, and the blue filled squares with upper and lower black lines represent the mean value (9.5 ± 2.3 mGy∙cm). Similarly, the red empty squares represent the size-specific dose estimate of each individual in the standard protocol group, and the red filled circles with upper and lower black lines represent the mean value (13.9 ± 4.8 mGy∙cm).

Fifty-one patients (mean age 65.9 years) were enrolled. The patient demographics, reduction of the radiation dose and the contrast agent volume are summarized in Table 1. The average scan interval was 204 days (SD = 128 days). The mean body mass index (BMI) was 24.6 (SD = 3.1). The contrast agent used in the reduction protocol group decreased by 24.4% compared to the standard protocol group. The mean portal phase size­specific dose estimate applying the patient’s transverse and anteroposterior diameters of the case group (9.5 ± 2.3 mGy∙cm) showed a 31.7% reduction than that of the standard protocol group (13.9 ± 4.8 mGy∙cm) (*p* < 0.001) (Fig 2). The mean tube current time product of the portal phase in the reduction protocol group (245.9 ± 69.5 mAs) increased by 41% compared to that in the standard protocol group (174.2 ± 64.0 mAs) (*p* < 0.001).

### Objective image analysis

**Fig 3:**
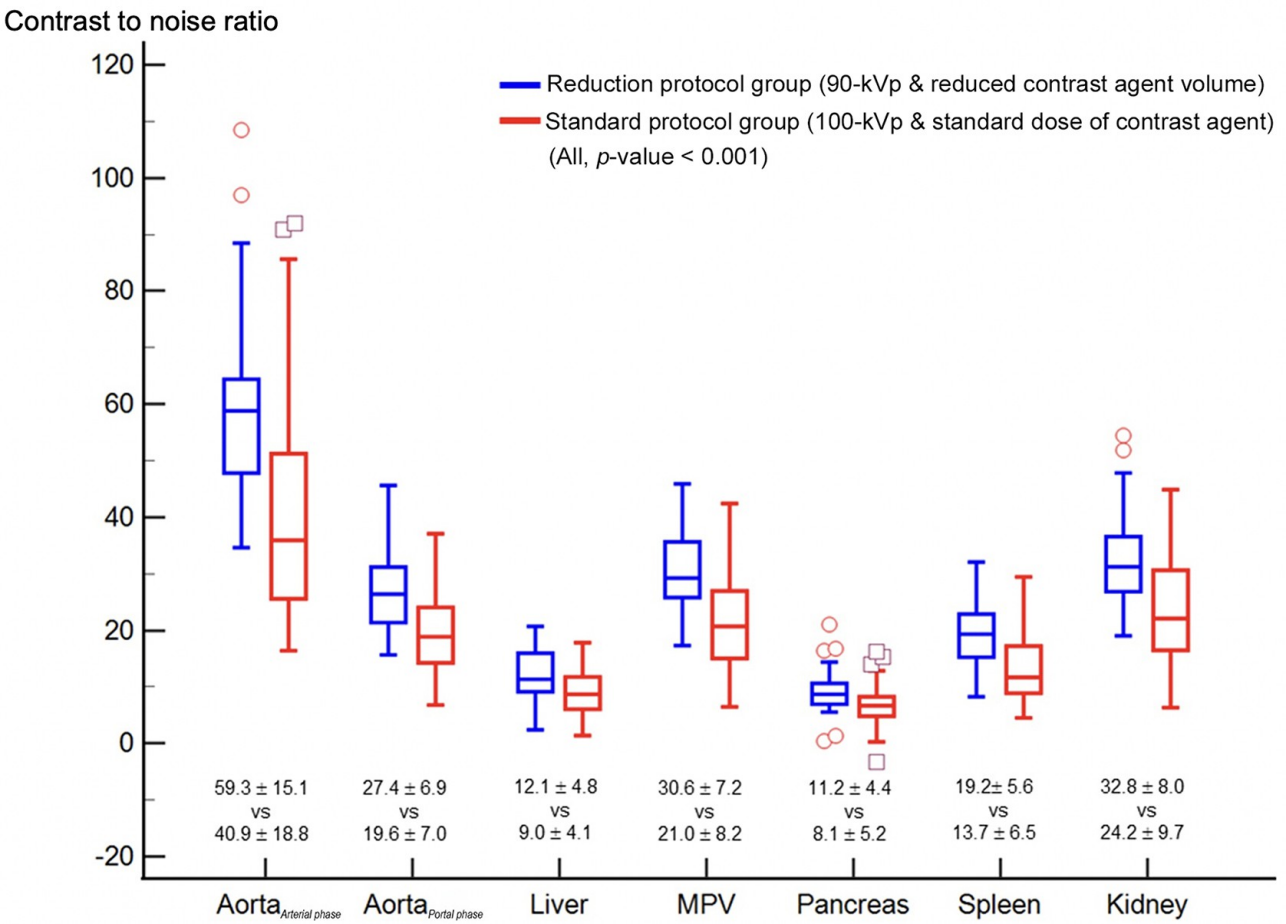
The box plot comparison of the contrast-to-noise ratio of each abdominal organs (Aorta_*Arterial phase*_ and Aorta_*Portal phase*_ are value measured on arterial and portal phase, respectively).

The SNR and CNR of each abdominal organ and the image noise as HU are summarized in Table 2, and the CNR is shown in Fig 3. Image noise of the reduction protocol group (5.4 ± 0.9) was significantly reduced compared to that of the standard protocol group (6.8 ± 2.4) by 20.6% (*p* < 0.001). The SNR of the psoas muscle showed no significant difference between the groups (*p* = 0.135). The SNR and CNR of the aorta, liver, MPV, pancreas, spleen, and kidney were significantly higher in the reduction protocol group (*p* < 0.001).

**Table 2:** Paired *t*-test results of objective image quality.

	Reduction protocol group (90-kVp tube voltage & reduced contrast agent volume)	Standard protocol group (100-kVp tube voltage & standard dose of contrast agent)	*p-*value
Image noise (arterial phase, HU)	5.3 ± 1.1	6.2 ± 1.7	< 0.001
Image noise (portal phase, HU)	5.4 ± 0.9	6.8 ± 2.4	< 0.001
Signal to Noise Ratio			
Aorta (arterial phase)	81.1 ± 23.8	48.9 ± 19.0	< 0.001
Aorta (portal phase)	39.7 ± 8.7	29.1 ± 9.5	< 0.001
Liver (portal phase)	24.4 ± 6.4	18.6 ± 6.7	< 0.001
Main portal vein (portal phase)	43.0 ± 9.0	30.6 ± 10.7	< 0.001
Pancreas (arterial phase)	22.9 ± 6.0	17.0 ± 7.7	< 0.001
Pancreas (portal phase)	21.5 ± 4.8	16.3 ± 5.6	< 0.001
Spleen (portal phase)	29.3 ± 6.3	21.5 ± 7.4	< 0.001
Renal cortex (portal phase)	45.1 ± 9.8	33.7 ± 12.2	< 0.001
Psoas muscle (arterial phase)	11.7 ± 2.8	10.8 ± 2.6	0.213
Psoas muscle (portal phase)	11.4 ± 3.2	10.4 ± 3.2	0.135
Contrast to Noise Ratio			
Aorta (arterial phase)	59.3 ± 15.1	40.9 ± 18.8	< 0.001
Aorta (portal phase)	27.4 ± 6.9	19.6 ± 7.0	< 0.001
Liver (portal phase)	12.1 ± 4.8	9.0 ± 4.1	0.001
Main portal vein (portal phase)	30.6 ± 7.2	21.0 ± 8.2	< 0.001
Pancreas (arterial phase)	11.2 ± 4.4	8.1 ± 5.2	< 0.001
Pancreas (portal phase)	9.2 ± 3.5	6.7 ± 3.7	0.001
Spleen (portal phase)	19.2 ± 5.6	13.7 ± 6.5	< 0.001
Renal cortex (portal phase)	32.8 ± 8.0	24.2 ± 9.7	< 0.001

**Fig 4:**
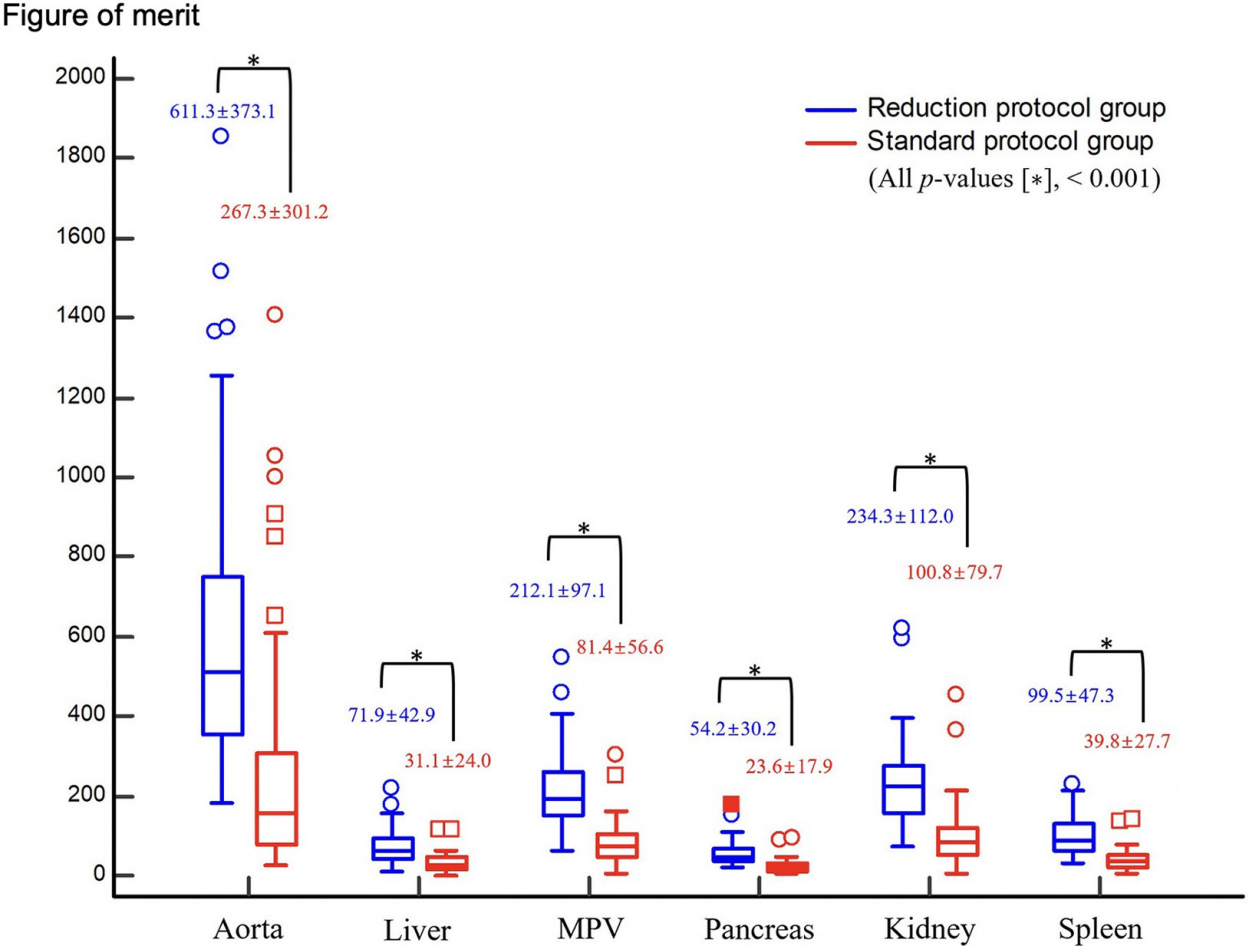
The box plot comparison of each organ’s figure of merit between the reduction and standard protocol groups. The figures of merit of the reduction protocol group were significantly higher than those of the standard protocol group (*p* < 0.001).

The figure of merit of image noise was no significant difference between the reduction protocol group (3.4 ± 1.5) and the standard protocol group (4.6 ± 5.7) (*p* > 0.1437). The figures of merit of aorta, liver, pancreas, kidney, and spleen in the reduction protocol group were significantly better than those in the standard protocol group (*p* < 0.001) (Fig 4).

### Subjective image analysis

**Fig 5:**
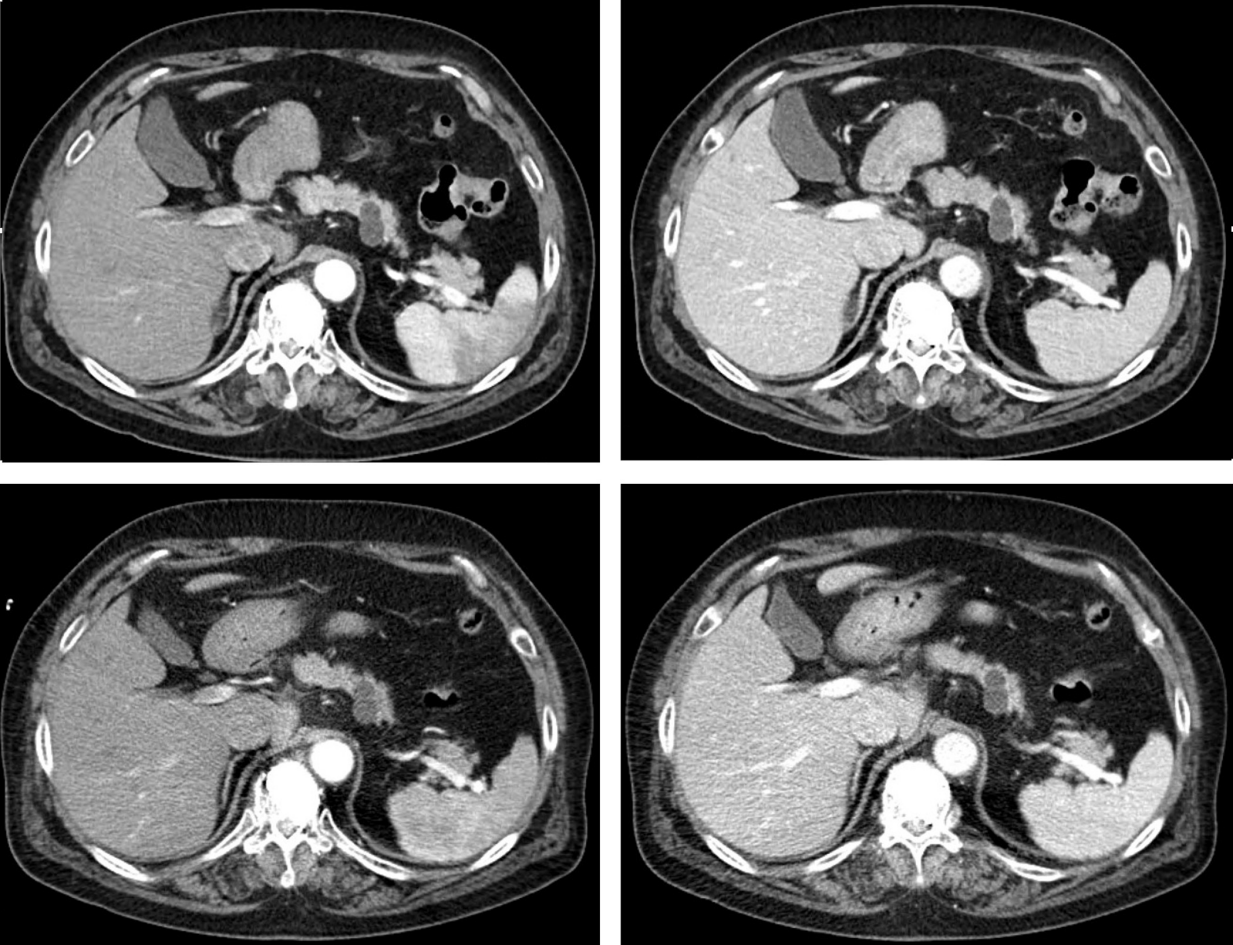
**A. A 53-year-old woman with a branch duct type intraductal papillary mucinous tumor in the pancreas tail**. A & B, Arterial and pancreatic parenchymal phase of computed tomography images of the reduction protocol group, respectively. C & D, Arterial and pancreatic parenchymal phase of computed tomography images of the standard protocol group, respectively. The reduction protocol images show reduction of beam hardening artefacts related kyphoplasty, and higher CNR of the pancreatic lesion based on the more enhancement of surrounding vessels and pancreatic parenchyma, compared to the standard protocol images. **B. A 53-year-old woman with a branch duct type intraductal papillary mucinous tumor in the pancreas tail**.

Reviewer 1 and reviewer 2 were off-site and on-site institution reviewers, respectively. Intraobserver and interobserver agreements were moderate to substantial (*k* = 0.4−0.8) (Table 3). The subjective image scores of both reviewers are summarized in Table 4. Except for lesion conspicuity, both reviewers judged that the image quality in the reduction protocol group was better than that in the standard protocol group. For lesion conspicuity, 40 hepatic cysts (8 ± 3 mm), 30 pancreatic cystic lesions (12 ± 7 mm), and five hepatic hemangiomas (15 ± 8 mm) were evaluated. All lesions were detected by both reviewers on both groups. Lesion conspicuity was measured to be higher than 4.0 in both groups, but there was no statistically significant difference. Fig 5 shows the representative case of the pancreatic cystic neoplasm scanned by the reduction protocol group (Fig 5A and 5B) and the standard protocol group (Fig 5C and 5D) with 172 days interval.

A & B, Arterial and pancreatic parenchymal phase of computed tomography images of the reduction protocol group, respectively. C & D, Arterial and pancreatic parenchymal phase of computed tomography images of the standard protocol group, respectively. The reduction protocol images show reduction of beam hardening artefacts related kyphoplasty, and higher CNR of the pancreatic lesion based on the more enhancement of surrounding vessels and pancreatic parenchyma, compared to the standard protocol images. **C. A 53-year-old woman with a branch duct type intraductal papillary mucinous tumor in the pancreas tail**

A & B, Arterial and pancreatic parenchymal phase of computed tomography images of the reduction protocol group, respectively. C & D, Arterial and pancreatic parenchymal phase of computed tomography images of the standard protocol group, respectively. The reduction protocol images show reduction of beam hardening artefacts related kyphoplasty, and higher CNR of the pancreatic lesion based on the more enhancement of surrounding vessels and pancreatic parenchyma, compared to the standard protocol images. **D. A 53-year-old woman with a branch duct type intraductal papillary mucinous tumor in the pancreas tail**

A & B, Arterial and pancreatic parenchymal phase of computed tomography images of the reduction protocol group, respectively. C & D, Arterial and pancreatic parenchymal phase of computed tomography images of the standard protocol group, respectively. The reduction protocol images show reduction of beam hardening artefacts related kyphoplasty, and higher CNR of the pancreatic lesion based on the more enhancement of surrounding vessels and pancreatic parenchyma, compared to the standard protocol images.

**Table 3:** Intra-rater and inter-rater agreement of subjective image quality score.

	Intra-rater agreement (ICC)	Inter-rater agreement (kappa)
Subjective image noise	0.7 (0.6−0.8)	0.5 (0.4−0.6)
Visibility of small structures (Visibility of peripheral hepatic vessels)	0.8 (0.7−0.8)	0.5 (0.4−0.7)
Beam hardening or streak artifact	0.5 (0.4−0.7)	0.4 (0.3−0.6)
Lesion conspicuity	0.8 (0.7−0.8)	0.4 (0.3−0.5)
Overall diagnosis confidence	0.7 (0.5−0.8)	0.5 (0.3−0.7)

Numbers in parentheses are 95% confidence interval.

**Table 4:** Paired comparison of subjective image quality by the Wilcoxon signed-rank test.

	Reviewer 1			Reviewer 2		
	Reduction protocol group	Standard protocol group	*p*-value	Reduction protocol group	Standard protocol group	*p*-value
Subjective image noise	4.3 ± 0.5	3.4 ± 0.7	< 0.001	3.5 ± 0.5	2.7 ± 0.6	< 0.001
Visibility of small structures (Visibility of peripheral hepatic vessels)	3.6 ± 0.5	3.1 ± 0.7	< 0.001	3.6 ± 0.5	3.1 ± 0.6	< 0.001
Beam hardening or streak artifact	4.9 ± 0.3	4.8 ± 0.4	0.086	4.3 ± 0.5	3.9 ± 0.7	0.002
Lesion conspicuity	4.7 ± 0.6	4.8 ± 0.4	0.261	4.1 ± 1.0	4.0 ± 1.1	0.352
Overall diagnostic confidence	4.9 ± 0.3	4.5 ± 0.6	< 0.001	4.6 ± 0.8	3.9 ± 0.9	< 0.001

## Discussion

As a result, the mean amount of contrast agent in the 90-kVp tube voltage group was reduced by 24.4% compared to the standard amount in the 100-kVp tube voltage group. In addition, despite a 41.2% increase in tube current time product in the 90-kVp tube voltage group compared to the 100-kVp tube voltage group, the size-specific dose decreased by 31.7%. Thus, contrary to the concern of the decrease in tube voltage and the increase in tube current time product, decreased amount of total contrast agent and advanced iterative reconstruction algorithm achieved decreased radiation dose with significantly better objective image quality in the 90-kVp tube voltage group. The subjective image quality results were also validated by objective image quality analysis. Each subjective image quality score was significantly higher in the 90­kVp tube voltage scan images than 100­kVp tube voltage scan images, except for the lesion conspicuity score. Subjective image quality was highly scored not only by the on-site institution reviewer but also by the off­site institution reviewer, who was not familiar with on-site institution images. The detection of low-density lesions in the liver and pancreas may be limited owing to the deterioration of the image quality in low-tube-voltage images. The lesion conspicuity score showed no significant difference between the case and standard protocol groups; it was measured at an average of 4 points or more, so there will be less impact on lesion detection. Based on the effect of improving the SNR and CNR of the image and the reduction of image noise and artefacts, the overall diagnostic confidence was higher in the reduction protocol group than in the standard protocol group.

The k-edge of iodine is 33.2 KeV and it is closer to 90 kVp than to 100 kVp, which means the attenuation of iodine contrast is substantially higher at 90 kVp than at 100 kVp. Therefore, theoretically, similar image quality can be obtained even with a relatively small amount of contrast agent in 90-kVp tube voltage CT when compared to 100-kVp tube voltage CT. Buls et al. reported that when 80­kVp tube voltage scan was applied, a similar contrast enhancement effect could be achieved even with 30% less contrast agent compared to 120­kVp tube voltage scan for abdominopelvic CT scan [27]. To minimize the degradation of image quality, such as unexpected deterioration of the contrast enhancement effect, we performed PDCT with 90­kVp tube voltage and contrast medium reduced by 30% than the standard amount because CT scans in humans are greatly affected by BMI and body habitus. This study proved that the 90­kVp low-voltage scan simultaneously reduced the radiation dose and the total amount of contrast agent without deterioration of the image quality compared to the 100­kVp tube voltage scan.

The reduction in the radiation dose was mainly dependent on the tube potential. The radiation dose is proportional to the square of the tube potential [3]. Reducing the tube voltage from 100-kVp to 80­kVp reduces the radiation dose by a factor of 1.5 [28]. In a previous study, there was the achievement of a 13.3% radiation dose reduction using 100­kVp tube voltage with IR compared with 120­kVp tube voltage in abdomen-pelvic CT [29]. As the tube current is directly proportional to the radiation dose, a 50% reduction in tube current results in a 50% reduction in radiation dose. The decrease in image quality owing to a decrease in the X-ray tube voltage is compensated by an increase in the tube current. In fact, following the result of the study, as the tube voltage decreased by 10­kVp, the total radiation dose finally decreased by 31.7%, although the tube current time product increased by 41.2%.

Marin et al. reported radiation dose reduction and improved imaging features related to contrast enhancement effects, such as CNR of the aorta, pancreas, portal vein, and pancreas to tumor and lesion conspicuity [6]. Even though we reduced the contrast medium dose to approximately 30% of the standard amount based on patient body weight, the image quality was significantly improved compared to the standard protocol group. The difference of image quality may have resulted because, the reduction protocol group’s equipment was updated, and the latest versions of IR and high tube current techniques were installed. The reduction protocol group showed a higher mean tube current time product and lower image noise than the standard protocol group. Low tube voltage CT imaging has the disadvantage of increasing image noise, but the overall image quality is improved by removing the image noise through the IR algorithm instead of the classical image reconstruction algorithm known as filtered back-projection [30]. The IR applied in the reduction protocol group was a more advanced algorithm than that in the standard protocol group, and as a result, the image noise of the reduction protocol group was significantly lower than that of the standard protocol group by 20.6%. With compensation by high tube current modulation and the latest IR technique, a 90­kVp tube voltage scan improved both objective and subjective image quality without impairing lesion detection and diagnostic confidence despite the reduction in tube voltage and the total amount of contrast agent.

This study has several limitations. First, only a few patients with a BMI of 30 or higher were included to minimize the image quality degradation. Since patients with a BMI of 30 or higher are usually selected with a tube voltage of 100-kVp or 120-kVp, the patients included in the study were BMI of less than 30 and the BMI was distributed within a certain range. Second, there was a difference in the equipment. The CT used in the reduction group was introduced one generation later than that of the standard protocol’s equipment; thus, the IR algorithm and X-ray tube technology were more advanced. However, since designed as retrospective study, inevitable difference could not be avoided. Nevertheless, the study showed that the combination of advanced IR and advanced x-ray tube technology such as low kVp and high tube current modulation can simultaneously reduce radiation exposure and reduce the amount of contrast medium used, compared to the conventional PDCT scan protocol that has been widely used. Moreover, the fact that 90 kVp CT and high tube current modulation are not widely available in all hospitals can be another limitation, but despite this, there is significance in reducing radiation exposure and contrast agent dosage in PDCT while adhering to the ALARA (as low as reasonably achievable) principle without deterioration of image quality. Additionally, to our knowledge, it is the first study to evaluate the image quality of abdominal solid organs and the radiation dose reduction with low tube voltage CT scanned with reduced amount of the contrast material in PDCT, thus, our study has own originality. Third, high-contrast lesions of the pancreas and liver, such as hepatic hemangioma, hepatocellular carcinoma, and neuroendocrine tumors of the pancreas, could not be evaluated because there were few or no forementioned lesions in the sampling. However, we could estimate the possibility of evaluating hypervascular disease to be probably high, considering attenuation of the vessels. Fourth, the results were dependent on the specific CT manufacturer. For more spillover effect, the future study should compare CT scanners of various vendors, which are enabled with low kVp tube voltage scan compensated by high tube current modulation with or without advanced IR.

In conclusion, the combination of 90­kVp tube voltage, IR, and high tube current achieved a reduction of the total amount of contrast medium by 25% and the radiation dose by 31.7% without deterioration of image quality and diagnostic reliability compared with 100­kVp tube voltage with standard amount of contrast agent in PDCT.

## Supporting information

S1 Table STROBE statement—checklist of items that should be included in reports of case-control studies. (DOC)

S2 Table The contrast agent administration adjustment table of the 90-kVp pancreas dynamic CT protocol. (DOCX)

S3 Table Summary of a 5-point score of subjective image quality analysis. (DOCX)
