# Supplementary material for: Reducing contrast-agent volume and radiation dose in CT with 90­kVp tube voltage, high tube current modulation, and advanced iteration algorithm
Source: PLoS One. 2023 Jun 15;18(6):e0287214. doi: 10.1371/journal.pone.0287214 (PMC10270572; doi:10.1371/journal.pone.0287214)
Supplement: S2 Table — (DOCX) [file pone.0287214.s002.docx]

**Supplemental Table 2. The contrast agent administration adjustment table of the 90-kVp pancreas dynamic CT protocol**.

| Body weight (kg) | Contrast agent volume (ml) | Flow rate (ml/sec) |
| --- | --- | --- |
| 40 | 60 | 2.0 |
| 45 | 68 | 2.0 |
| 50 | 75 | 2.0 |
| 55 | 83 | 2.1 |
| 60 | 90 | 2.3 |
| 65 | 98 | 2.4 |
| 70 | 105 | 2.6 |
| 75 | 113 | 2.8 |
| 80 | 120 | 3.0 |
| 85 | 128 | 3.2 |
| 90 | 135 | 3.4 |
| 95 | 143 | 3.6 |
| 100 | 150 | 3.8 |
| 105 | 158 | 3.9 |
| 110 | 165 | 4.1 |
| 115 | 173 | 4.3 |
| 120 | 180 | 4.5 |
