# Supplementary material for: Reducing contrast-agent volume and radiation dose in CT with 90­kVp tube voltage, high tube current modulation, and advanced iteration algorithm
Source: PLoS One. 2023 Jun 15;18(6):e0287214. doi: 10.1371/journal.pone.0287214 (PMC10270572; doi:10.1371/journal.pone.0287214)
Supplement: S3 Table — (DOCX) [file pone.0287214.s003.docx]

Supplemental Table 3. Summary of a 5-point score of subjective image quality analysis.

| Subjective image noise | |
| --- | --- |
| 1 | unacceptable image noise |
| 2 | above average |
| 3 | average |
| 4 | less than average |
| 5 | minimal image noise |
| Visibility of small structure (peripheral hepatic vessels) | |
| 1 | unacceptable visualization (peripheral hepatic vessels and bifurcations are unidentified) |
| 2 | suboptimal visibility (peripheral hepatic vessels are unclear or tram-track appearance, but, bifurcation is identified) |
| 3 | average visibility (peripheral heaptic vessels and bifurcation shows blurring) |
| 4 | above average visibility (one of peripheral heaptic vessels or bifurcation show clear identification without blurring) |
| 5 | excellent visibility (Both peripheral heaptic vessels and bifurcation show clear identification without any blurring) |
| Beam hardening or streak artifact | |
| 1 | artifacts affecting the interpretation of lesions or organs of interests, diagnostic decision is impossible |
| 2 | artifacts affecting the interpretation of lesions or organs of interests, diagnostic decision is possible |
| 3 | moderate artifacts slightly interfering with diagnostic decision |
| 4 | mild artifacts not interfering with diagnostic decision |
| 5 | complete absence of artifact |
| Lesion conspicuity | |
| 1 | imperceptible lesion |
| 2 | lesion margin is unclear |
| 3 | 25-50% of lesion margin is clear |
| 4 | 50-75% of lesion margin is clear |
| 5 | more than 75% lesion margin is clear |
| Overall diagnostic confidence | |
| 1 | unacceptable diagnosis |
| 2 | sub-diagnostic confidence |
| 3 | average confidence |
| 4 | better than average |
| 5 | completely confident |
